# Phytohormones as Important Biologically Active Molecules – Their Simple Simultaneous Detection

**DOI:** 10.3390/molecules14051825

**Published:** 2009-05-15

**Authors:** Vaclav Diopan, Vojtech Adam, Ladislav Havel, Rene Kizek

**Affiliations:** 1 Department of Plant Biology, Faculty of Agronomy, Mendel University of Agriculture and Forestry, Zemedelska 1, CZ-613 00 Brno, Czech Republic; E-mails: diopan@email.cz (V.D.), lhavel@mendelu.cz (L.H.); 2 Department of Chemistry and Biochemistry, Faculty of Agronomy, Mendel University of Agriculture and Forestry, Zemedelska 1, CZ-613 00 Brno, Czech Republic; E-mails: ilabo@seznam.cz (V.A.); 3 Department of Animal Nutrition and Forage Production, Faculty of Agronomy, Mendel University of Agriculture and Forestry, Zemedelska 1, CZ-613 00 Brno, Czech Republic; E-mail: ilabo@seznam.cz (V.A.)

**Keywords:** phytohormones, liquid chromatography, UV-VIS detection, flax, mass spectrometry

## Abstract

Phytohormones, their functions, synthesis and effects, are of great interest. To study them in plant tissues accurate and sensitive methods are required. In the present study we aimed at optimizing experimental conditions to separate and determine not only plant hormones but also their metabolites, by liquid chromatography coupled with a UV-VIS detector. The mixture we analyzed was composed of benzyladenine, kinetin, *trans*-zeatin, *cis*-zeatin, dihydrozeatin, *meta*-topolin, *ortho*-topolin, α-naphthalene acetic acid, indole-3-acetic acid, *trans*-zeatin-7-glucoside, *trans*-zeatin-*O*-glucoside, *trans*-zeatin-9-riboside, *meta*-topolin-9-riboside and *ortho*-topolin-9-riboside. We measured the calibration dependences and estimated limits of detection and quantification under the optimal chromatographic conditions (column: Polaris C_18_; mobile phase: gradient starting at 2:98 (methanol:0.001% TFA) and was increasing to 55:45 during twenty minutes, and then decreasing for 10 min to 35:65, flow rate: 200 µL·min^-1^, temperature: 50 °C, wavelength: 210 nm). The detection limits for the target molecules were estimated as tens of ng per mL. We also studied the effect of flax extracts on the phytohormones’ signals. Recovery of aliphatic and aromatic cytokinins, metabolites of cytokinins and auxins were within the range from 87 to 105 %. The experimental conditions were tested on a mass selective detector. In addition we analysed a commercial product used for stimulation of roots formation in cuttings of poorly rooting plants. The determined content of α-naphthalene acetic acid was in good agreement with that declared by the manufacturer.

## Introduction

In plants, a group of chemically varied compounds called phytohormones (plant growth regulators) regulates physiological processes. The main groups of phytohormones are auxins, cytokinins, brassinosteroids, gibberellins, jasmonic acid and abscisic acid. Within a specific group phytohormones are further subdivided according their chemical structures. Cytokinins induce cell division, morphogenesis (shoot and bud initiation and formation, growth of lateral buds) and delay the senescence of their tissues. Synthetic cytokinins are used in plant production and in cultivation processes. Apical dominance is controlled by the concentration gradient of auxins, another group of phytohormones [1,2,3]. These phytohormones are also responsible for the tropism, elongation and roots formation. The inhibiting hormones play role in dormancy maintaining in buds and seeds and their level increases under stress [4,5].

Except for their origonal structures, all phytohormones are present as various derivatives, e.g. degradation metabolites, transport, (in)activated or storage forms, especially conjugates with sugars or amino acids. Some of these derivatives often have the same biological activity as the free hormones, so their concentrations must be taken into account for an accurate estimation of their effects [6]. Moreover, several biological effects of phytohormones are induced by cooperation of more than one phytohormone. 

**Figure 1:**
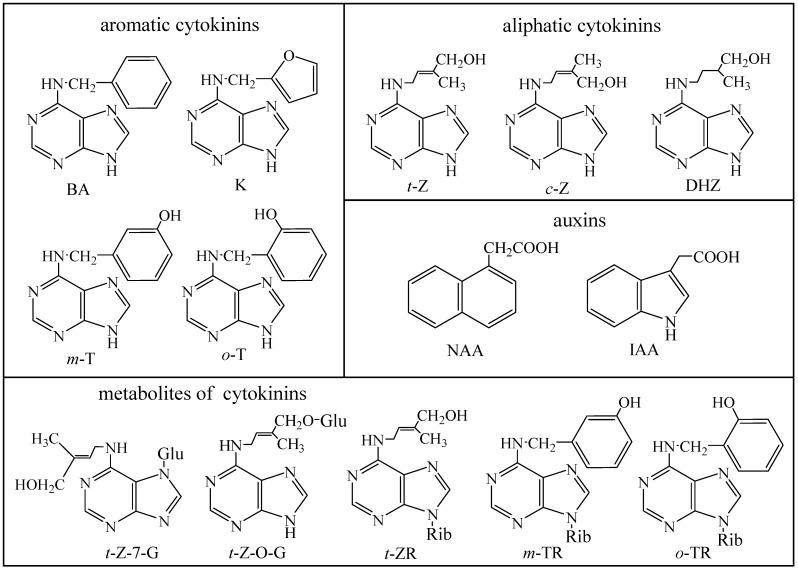
Chemical formulas of the phytohormones. BA = benzyladenine, K – kinetin, *t*-Z = *trans*-zeatin, *c*-Z = *cis*-zeatin, DHZ = dihydrozeatin, m-T = *meta*-topolin, o-T = *ortho*-topolin, NAA = α-naphthalene acetic acid, IAA = indole-3-acetic acid, *t*-Z-7-G = *trans*-zeatin-7-glucoside, *t*-Z-*O*-G = *trans*-zeatin-O-glucoside, *t*-ZR = *trans*-zeatin-9-riboside, *m*-TR = *meta*-topolin-9-riboside and *o*-TR = *ortho*-topolin-9-riboside. Rib = ribose, Glu = glucose.

Thin-layer chromatography was the first technique used for rapid separation and determination of phytohormones [7,8,9]. Nowadays, high performance liquid chromatography (HPLC) coupled with various types of detectors is the most commonly used technique for separation of phytohormones. As a detector, UV spectrometers, mass spectrometers or electrochemical detectors are used [10,11,12,13,14,15,16]. Coupling of HPLC with mass spectrometry is advantageous, as shown by Prinsen *et al*., Novak *et al*. and Chiwocha *et al*., who determined several phytohormones simultaneously with a detection limit under 1 pmol [11,17,18,19,20]. Besides liquid chromatography, capillary electrophoresis was also utilized for the analysis of phytohormones [21,22,23,24]. Papers reporting on determination of phytohormones are typically aimed at one group of these substances, such as cytokinins, auxins and their metabolites, however, the trend in hormone physiology goes towards hormone profiling and screening for large numbers of compounds [7,25,26,27]. Therefore, the main aim of this work was to optimize conditions for simultaneous HPLC-UV separation of fourteen phytohormones from various phytohormones groups (Figure 1). Then a real sample of a commercial product used for stimulation of root formation in cuttings of poorly rooting plants was studied using the optimized conditions.

## Results and Discussion

Due to chemical diversity of the phytohormones as a result of their substituent groups on the one hand and due to the chemical similarity of these substances within a particular group on the other hand, it was necessary to optimize several experimental conditions (wavelength, column, temperature, mobile phase) for their sensitive and effective simultaneous detection.

### Wavelength

**Figure 2:**
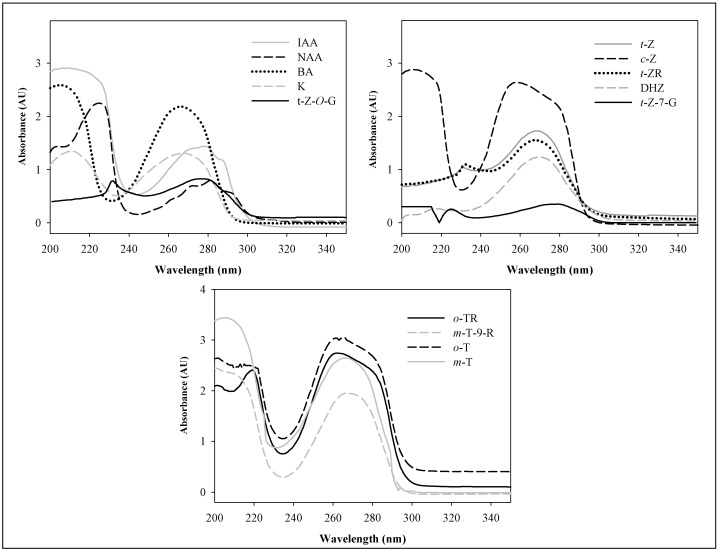
Molecular absorption UV spectra of the phytohormones. Phytohormone concentration: 20 μg.mL^-1^ optical path length: 1 cm.

The absorption spectra of the target molecules were measured over the UV range from 200 to 300 nm. The absorption maxima of the each phytohormone are different (Figure 2). If we consider demands on simultaneous detection of these compounds, two absorption maxima, 210 nm and 270 nm, can be used. We used z detector setting at 210 nm in the following experiments. Under this wavelength, the absorption coefficients of *t*-Z, DHZ, *t*-ZR and zeatin glucosides are lower in comparison with 270 nm, but similar or even higher for the other ones.

### Column 

Low molecular weight biologically active compounds are readily separable using reversed phase high performance liquid chromatography [28,29,30,31,32]. The most commonly used reversed phase is octadecyl C_18_. The properties of the columns from different suppliers are not same because different technologies are used in the preparation of the support materials and the actual packing of the columns. In this work, C_18_ columns from three various suppliers (Aquasil, Phenomenex and Polaris) were tested. Using of two columns (Aquasil and Phenomenex) under similar conditions (2:98 methanol:water, flow rate: 200 µL·min^-1^, temperature: 25°C, wavelength: 210 nm) resulted in the shorter retention times, leading to co-elution of the compounds. Therefore we analysed the phytohormones on the Polaris column in the subsequent experiments.

### Temperature

The temperature is one of the more important separation parameters. Temperature accelerates mass transfer in the column and lowers the diffusion coefficient of the mobile phase. The separation under higher temperatures leads to higher resolution and shorter elution times. We tested the influence of temperature within the interval from 20 to 60 °C (in 5 °C steps, not shown). Based on the results obtained the temperature 50 °C was chosen, because the best resolution and the shortest measurement time were achieved. At higher temperatures co-elution of *c*Z with DHZ and *m*-TR with K occurred.

### Mobile phase and the effect of trifluoroacetic acid

**Figure 3:**
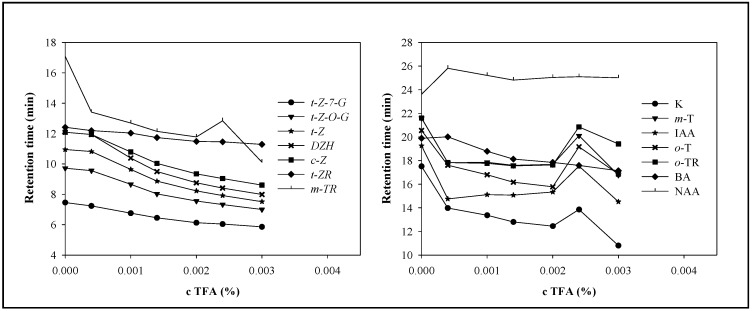
The dependence of reduced retention time on TFA concentration. The best resolution was obtained at TFA concentration 0.001%. The concentrations of individual phytohormones were chosen with respect to similar peaks height and were within the interval from 6 to 20 μg·mL^-1^. The detection was carried out at 210 nm. Linear gradient from 2:98 methanol:TFA (0 min.) up to 85:15 methanol:TFA (40 min.). Mobile phase flow rate of 250 μL·min^-1^.

A mobile phase composed of methanol and trifluoroacetic acid (TFA) was used. In order to achieve easily repeatable measurements no buffer components that might shorten the lifetime of the column was used. TFA (0; 0.00040; 0.001; 0.0014; 0.002; 0.0024; 0.003%, *v*/*v*) was tested. TFA addition affected the retention time, symmetry and peaks resolution by changing the pH level and the ionization of the phytohormones. The changes in retention times depending on TFA concentration are shown in Figure 3. TFA had considerable effect on the symmetry and resolution of the phytohormones’ peaks. We assume the mechanism of action of TFA on phytohormones to be similar to that on peptides. According these presumptions, chemically similar cytokinins should be determined with better resolution and symmetry. We observed that the prevailing effect of an increasing TFA concentration on retention times was their reduction, due to ion-pairing of the TFA resulting in the enhancement of the polarity of the target molecules, which confirmed our presumption. Only NAA increased its retention time, which probably relates with the fact that TFA inhibited ionisation and, thus, prolonged the time of interaction of this phytohormone with the stationary phase. The highest reduction of the retention time (by more than 7 minutes comparing values measured under null and 0.003% TFA) occurred for *m*-TR and K. It clearly follows from the results obtained that the best resolution was obtained at 0.001% TFA. All phytohormones’ peaks were well separated and symmetrical.

**Figure 4:**
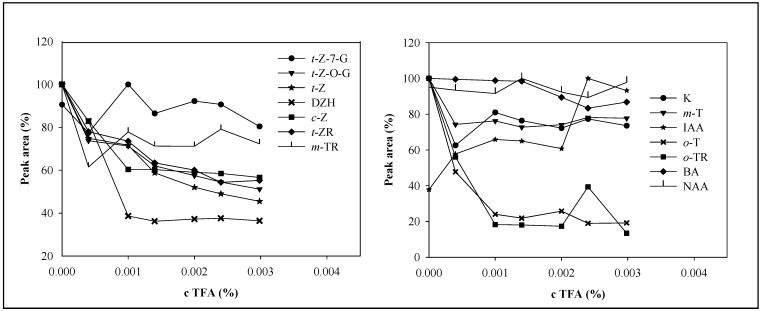
Dependence of phytohormones’ peak areas on TFA concentration. Chromatographic conditions were the same as in Figure 3.

The TFA concentration affected also the area of the phytohormones’ peaks (Figure 4). The increasing TFA concentration decreased the response. The major decrease occurred within the interval from 0.00040 to 0.001% TFA. A higher concentration of TFA did not decrease the signal much. In the case of DHZ, *o*-T and *o*-TR, a sharp decrease of peak areas was observed. On the contrary, the response of IAA increased with increasing TFA concentration. The NAA peak area did not change much. Although TFA had adverse effect on peak area of the studied compounds, except for IAA, its presence was essential for good separation. Therefore, we selected a mobile phase consisting of methanol and 0.001% TFA for the subsequent experiments.

### Gradient profile and flow rate optimisation

Gradient elution is used for separation of compounds with different affinity towards a stationary phase. Application of gradients enables separation of more complicated matrices, and eventually a reduction of analysis time. In spite of the fact that it was possible to separate well all phytohormones of interest isocratically, we tested a ternary gradient to improve resolution of *c*-Z and DHZ (tR = 12.09 and 12.27 min, respectively). The optimized gradient is shown in Table 1. Resolution of the peaks was improved and time of analysis was shortened by about six minutes, compared to isocratic elution with 20 % MeOH.

**Figure 5:**
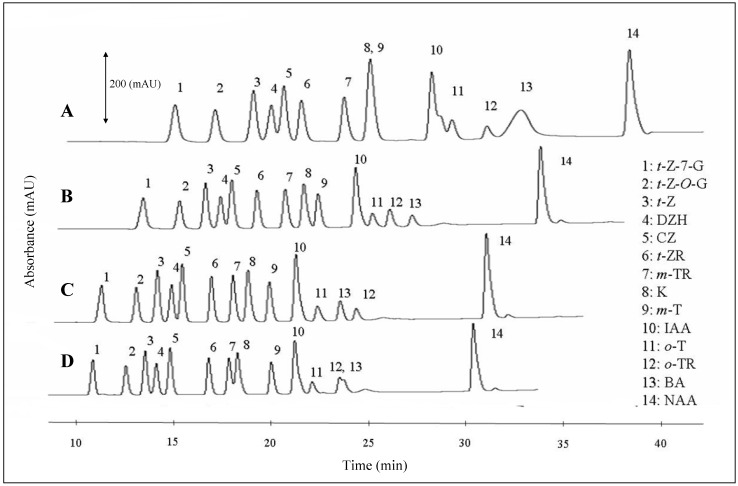
Typical HPLC-UV chromatograms of phytohormones measured at four various flow rates: A – 100 μL·min^-1^, B – 150 μL·min^-1^, C – 200 μL·min^-1^ and D – 250 μL·min^-1^. The best separation was achieved at 200 μL·min^-1^.

Flow rates of the mobile phase within the interval from 100 to 250 µL·min^-1^ was another tested parameter, The changes in chromatograms are shown in Figure 5. It follows from the results obtained that the most suitable flow rate for simultaneous detection of phytohormones is 200 μL·min^-1^. Lower or higher flow rates resulted in overlapping signals. At lower flow rates, the K peak overlapped the *m*-T peak and the IAA one overlapped the *o*-T one. At higher flow rates, the peaks of *o*-TR and BA were overlapped (Figure 5). 

Under the optimal chromatographic conditions (column: Polaris C_18_, mobile phase: Table 1, flow rate: 200 µL·min^-1^, temperature: 50 °C, wavelength: 210 nm) we measured the calibration dependences and estimated limits of detection and quantification (Table 2). The concentration interval, in which analytical data have been obtained, was different, depending on sensitivity of the method to certain compounds. Compounds providing the highest responses (IAA and NAA) were measured within the interval from 0.8 to 6.25 µg·mL^-1^. The least detectable compound (*t*-Z-7-G) had an interval from 2.8 to 22 µg·mL^-1^.

A study of the effects of the heavy metal ions on phytohormones could provide valuable data for processes using biological systems (plants, bacteria, fungi) for the remediation of soil and water contaminated with toxic metals and other toxic substances. These methods are environmentally friendly alternatives to industrially applied processes. The advantage of these approaches in comparison with the conventional physico-chemical methods is their low price, minimum quantities of secondary waste produced and the possibility of removing contaminants from large areas without the need for extraction of the contaminated soil. Because of the bio-friendliness to the environment of these remediation approaches and their aesthetic value they are well accepted by the public. Flax seems to be one of the potential of plants suitable for remediation of heavy metals from contaminated soil, but there is still a lack detailed information on the biological changes caused by heavy metals, aside from well investigated plant heavy-metal-protective processes through synthesis of peptides rich in cysteine moieties [33,34,35,36,37,38,39,40]. In our experiments, we studied the effect of flax extracts on the phytohormones’ signals to use the results in subsequent biological experiments. Recovery of aliphatic and aromatic cytokinins, metabolites of cytokinins and auxins were evaluated with MeOH extract of flax (*Linum usitatissimum* L.) spiked with standards by HPLC-UV (210 nm). The extracts were assayed blindly and the phytohormones concentration was derived from the calibration curves. The spiking of hormones was determined as a standard measured without presence of real sample [41,42]. The results obtained are summarized in Table 2.

### Mass spectrometry of phytohormones

**Figure 6:**
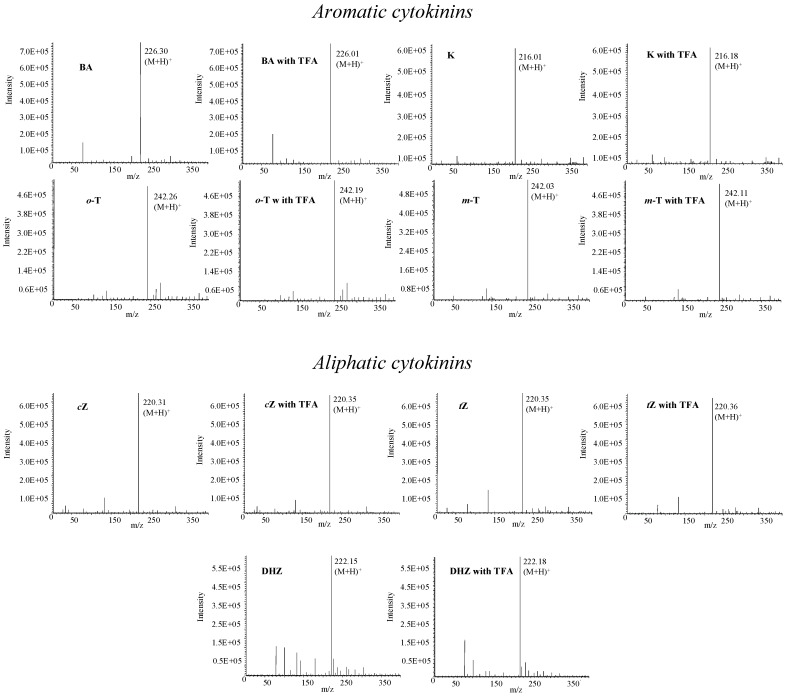

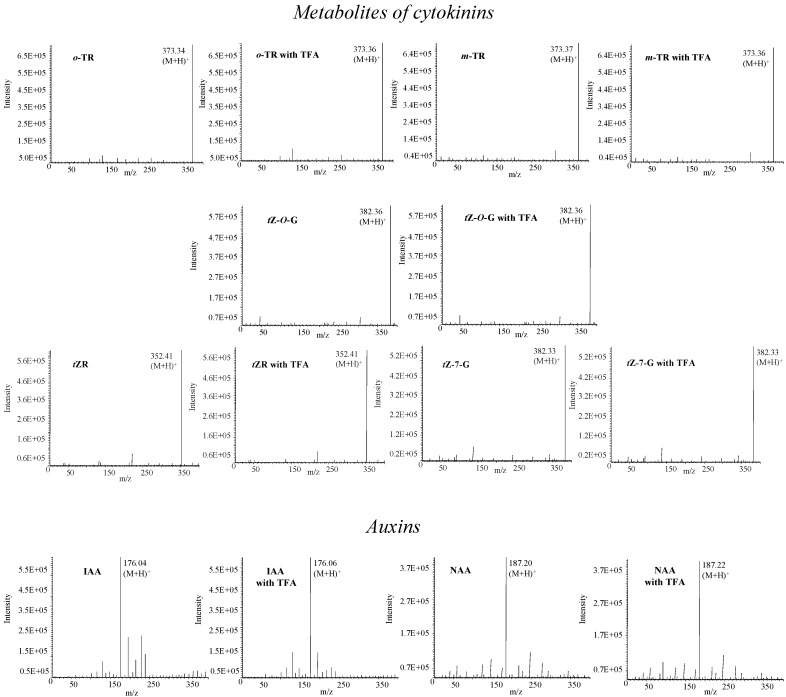
Mass spectra of phytohormones measured with or without addition of TFA in mobile phase. Concentration of TFA – 0.001%, mobile phase – methanol:water (50:50, v/v), capillary voltage 3.5 kV, drying gas temperature 250 °C, nebulizer pressure 5 MPa; drying gas flow 6 L·min^-1^; cone voltage – 20 V.

For ultrasensitive determination of target molecules HPLC is connected with modern and sensitive detectors, most commonly with mass spectrometers. There are several various types of mass spectrometers, including tandem mass spectrometers, which are very advantageous for analysis of overlapping signals due to different fragmentation of mother ions with the same mass. However, these instruments are costly. The method optimized in this study is suitable for detection of fourteen phytohormones. Considering the fact that mobile phase did not contain any buffer, the conditions can be applied on an instrument equipped with a mass detector. On the other hand, however, our mobile phase contained TFA, which, like other strong ion-pairing compounds, can also degrade the signals of target molecules [44]. Therefore, we studied the influence of TFA on the MS detection of phytohormones (Figure 6). Phytohormones (0.5 µg·mL^-1^) were detected using flow injection analysis with mass detection. Methanol:water (50:50, *v*/*v*) with or without addition of TFA (0.001 %, *v*/*v*) was used as a mobile phase. We detected molecular peaks (M+H)^+^ in all studied phytohormones under the above-mentioned experimental conditions. The intensity of molecular peaks was not influenced by the presence of TFA (decrease app. 2-4 % compared to non-TFA signals). We thus assume that any influence on the signals’ intensities due to the low TFA concentration used is negligible.

### Determination of phytohormones in growth stimulator

**Figure 7:**
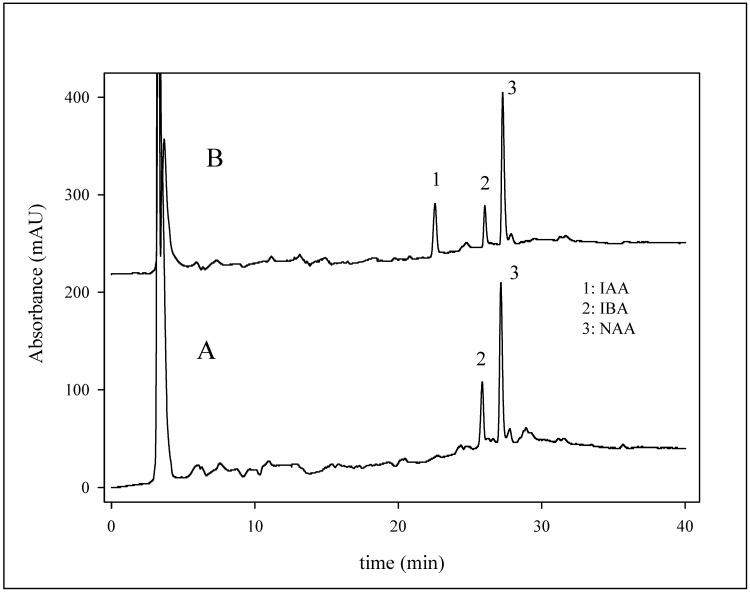
Chromatogram A shows analysis of fivefold diluted gelastim A. Chromatogram B shows analysis of the eightfold diluted gelastin A with standard addition of IAA and NAA (0.5 µg to one mL of the diluted sample). Chromatographic conditions were as follows: column: Polaris C_18_, mobile phase: Table 1, flow rate: 200 µL·min^-1^, temperature: 50 °C, wavelength: 210 nm, sample injection: 20 µL.

The proposed chromatographic method with UV detection was employed for analysis of the commercial product Gelastim A (Czech Republic) containing IAA, indole-3-butyric acid (IBA) and NAA, used for the stimulation of roots formation in cuttings of poorly rooting plants. According to the manufacturer, the product contains 9.5 mg·L^-1^ of α-naphthalene acetic acid (NAA). In addition, the mixture also contains 8-hydroxyquinoline sulphate (150 mg.L^-1^) for disinfecting the wounded surfaces of cuttings. HPLC measurement of the sample was carried out without any pre-treatment of the Gelastim A. The sample was diluted eight-fold with a mixture of methanol and water (1:1) prior to injection on the column. We determined two major signals in the chromatogram (Figure 7A). For identification of the compounds, measurement with standard addition of the growth regulators IAA, IBA (Sigma-Aldrich, USA) and NAA was carried out (Figure 7B). This addition demonstrated that the terminal signal in the chromatogram belongs to NAA, which was confirmed by a mass detector. According to calibration curve, we determined the NAA content as 9.3 ± 0.3 mg·L^-1^. This value is in good agreement with that declared by the manufacturer (9·5 mg.L^-1^).

## Experimental

### Chemicals

Phytohormones (*trans*-zeatin, *cis*-zeatin, dihydrozeatin, *trans*-zeatin-7-glucoside, *trans*-zeatin-9-riboside, *trans*-zeatin-O-glucoside, *meta*-topolin, *meta*-topolin-9-riboside, *ortho*-topolin, *ortho*-topolin-9-riboside, α-naphthalene acetic acid) purchased from Olchemim (Olomouc, Czech Republic) and indole-3-acetic acid, kinetin and 6-N-benzyladenine purchased from Sigma-Aldrich (St. Louis, USA) were of 98 % purity prior to HPLC analyses. Ultrapure Milli-Q (18 MΏ) water and methanol from Merck (Darmstadt, Germany) and trifluoroacetic acid (Sigma-Aldrich) were used as mobile phase.

The stock solutions of phytohormones (0.2 - 2 mg·mL^-1^) were prepared using methanol-water 50/50 (*w*/*w*) and stored in the dark at -20°C. The working solutions were prepared daily by diluting of the stock solutions. Stock *o*-TR solution was prepared daily due to its instability in methanol.

### High performance liquid chromatography with UV-VIS/MS detection (HPLC/UV/VIS/MS)

To separate the target molecules three reversed phase columns were used: Phenomenex C_18_, 250 × 2.1 mm, particle size 5 μm, MetaChem Polaris C_18_, 250 × 2.1 mm, particle size 5 μm and Thermoelectron Aquasil C_18_ 250 × 2.1 mm, particle size 5 μm. Chromatographic pump Rheos (Flux Instruments, Switzerland) connected with a HTS PAL (CTC Analytics, Sweden) autosampler was used. The instrument was controlled by Xcalibur software. UV-VIS detector SpectraSYSTEM UV 2000 (Thermo separation products Inc.) and mass detector Finnigan AQA (ThermoQuest) were used for detection of target molecules. The samples were injected by autosampler. Other chromatographic parameters were optimized and are shown in the “Results and Discussion” section.

### Plants

Flax (*Linum usitatissimum* L.) hybrid Viola was used in our experiments. Flax kernels were germinated on wet cellulose in Petri dishes at 23 ± 2 °C in dark. Germination was carried out in Versatile Environmental Test Chamber (MLR-350 H, Sanyo, Japan) for seven days with 12 h long daylight per day (maximal light intensity was about 100 μE.m^-2^s^-1^) at a temperature 21.5 – 22.5 °C and humidity 45 – 55 %. Cellulose was wetted twice a day to avoid its dessication. At the end of seven day long cultivation the seedlings were harvested and used in the subsequent experiments.

### Sample preparation

The harvested flax plants (app. 1 g of fresh weight) were extracted with 10 mL of methanol. The extract was purified with two solid-phase-extraction columns (WAT020515, Waters, USA). The extract was diluted eight times and spiked with phytohormones standards prior to evaluation of recovery.

### Descriptive statistics

Data were processed using Microsoft Excel® (USA). Results are expressed as mean ± standard deviation (S.D.), unless stated otherwise.

## Figures and Tables

**Table 1:** Gradient profile. The column was washed 80% methanol (*v*/*v*) for ten minutes and then methanol:water:water with 0.02% TFA (2:93:5) for ten minutes prior to each analysis.

min	MeOH (%)	Water (%)	Water with 0.02% TFA (%)
0	2	93	5
20	55	40	5
30	35	60	5

**Table 2:** Analytical parameters for HPLC-UV determination (n = 5).

Phytohormone	Regression	R^2^	^a^ LOD (ng/mL)	^b^ LOQ (ng/mL)	^c^ R.S.D. (%)	^d^ Recovery (%)
**Aliphatic cytokinins**						
*t*-Z	y = 91.193x - 10.217	1	60	200	4.4	96
*c*-Z	y = 88.334x - 3.5650	0.9996	50	170	5.5	98
DHZ	y = 54.361x - 10.050	0.9998	110	360	5.9	95
**Aromatic cytokinins**						
BA	y = 41.432x - 11.270	0.9988	130	430	4.6	87
K	y = 82.169x - 35.783	0.9991	70	230	4.5	98
*m*-T	y = 65.262x + 0.8680	0.9995	50	160	5.2	95
*o*-T	y = 56.429x - 33.103	0.9935	140	460	5.5	96
**Metabolites of cytokinins**						
*t*-ZR	y = 85.878x - 5.3040	0.9999	60	200	5.2	92
*t*-ZG	y = 34.709x - 6.4040	0.9999	150	500	5.5	105
*t*-ZOG	y = 42.958x - 9.007	1	130	430	5.8	103
*m*-TR	y = 77.946x - 47.529	0.9995	80	260	4.8	95
*o*-TR	y = 47.491x - 13.702	0.9988	120	400	4.5	98
**Auxins**						
IAA	y = 408.970x - 136.390	0.9945	20	70	4.8	97
NAA	y = 283.760x + 6.652	0.9985	20	70	3.9	99

^a^ Limit of Detection estimated (3 signal/noise, S/N) were calculated according to Long and Winefordner [43], whereas N was expressed as standard deviation of noise determined in the signal domain unless stated otherwise; ^b^ Limit of Quantification estimated as (10 S/N). [43]; ^c^ Relative Standard Deviation; ^d^ Recovery was estimated according to protocol mentioned below.
